# A novel mouse model carrying a gene trap insertion into the *Hmgxb4* gene locus to examine *Hmgxb4* expression in vivo

**DOI:** 10.14814/phy2.16014

**Published:** 2024-04-21

**Authors:** Liang Wang, Xiangqin He, Guoqing Hu, Jinhua Liu, Xiuhua Kang, Luyi Yu, Kunzhe Dong, Juanjuan Zhao, Aizhen Zhang, Wei Zhang, Michael W. Brands, Huabo Su, Zeqi Zheng, Jiliang Zhou

**Affiliations:** ^1^ Department of Cardiology The First Affiliated Hospital of Nanchang University Nanchang China; ^2^ Department of Pharmacology & Toxicology, Medical College of Georgia Augusta University Augusta Georgia USA; ^3^ Department of Respiratory Medicine The First Affiliated Hospital of Nanchang University Nanchang China; ^4^ Vascular Biology Center, Medical College of Georgia Augusta University Augusta Georgia USA; ^5^ Training Center Guangxi Medical College Nanning China; ^6^ Department of Physiology Medical College of Georgia Augusta Georgia USA

**Keywords:** arterial injury, gene expression, gene trap, *Hmgxb4*, β‐galactosidase activity

## Abstract

HMG (high mobility group) proteins are a diverse family of nonhistone chromosomal proteins that interact with DNA and a wide range of transcriptional regulators to regulate the structural architecture of DNA. HMGXB4 (also known as HMG2L1) is an HMG protein family member that contains a single HMG box domain. Our previous studies have demonstrated that HMGXB4 suppresses smooth muscle differentiation and exacerbates endotoxemia by promoting a systemic inflammatory response in mice. However, the expression of *Hmgxb4* in vivo has not fully examined. Herein, we generated a mouse model that harbors a gene trap in the form of a *lacZ* gene insertion into the *Hmgxb4* gene. This mouse enables the visualization of endogenous HMGXB4 expression in different tissues via staining for the β‐galactosidase activity of *LacZ* which is under the control of the endogenous *Hmgxb4* gene promoter. We found that HMGXB4 is widely expressed in mouse tissues and is a nuclear protein. Furthermore, the *Hmgxb4* gene trap mice exhibit normal cardiac function and blood pressure. Measurement of β‐galactosidase activity in the *Hmgxb4* gene trap mice demonstrated that the arterial injury significantly induces *Hmgxb4* expression. In summary, the *Hmgxb4* gene trap reporter mouse described here provides a valuable tool to examine the expression level of endogenous *Hmgxb4* in both physiological and pathological settings in vivo.

## 1 INTRODUCTION

HMG (high mobility group) proteins are a diverse family of nonhistone chromosomal proteins that nonspecifically change the conformational structure of DNA, or interact with a variety of transcription factors to modulate their functions (Hock et al., 2007; Travers, 2003). HMGXB4 (also known as HMG2L1) is a member of HMG protein family that contains a single HMG box domain (Yamada et al., 2003). HMGXB4 was originally shown to negatively regulate Wnt/β‐catenin signaling in Xenopus (Yamada et al., 2003). Through the yeast 2‐hybrid approach by using the potent transcription co‐factor myocardin as the bait, we demonstrated that HMGXB4 is a myocardin binding protein and can suppress smooth muscle differentiation by attenuating myocardin function (Zhou et al., 2010). Recently, our study further revealed a critical role of HMGXB4 in exacerbating endotoxemia by promoting a systemic inflammatory response in mice (He et al., 2021). Despite these discoveries made on this protein, its expression and function remain to be further studied.

In this study, we generated a mouse model harboring a gene trap *lacZ* gene inserted into the *Hmgxb4* gene locus. In these mice, staining for β‐galactosidase that is encoded by the *lacZ* gene and its expression is under control of the endogenous *Hmgxb4* gene promoter, can be used as a reporter for monitoring the transcriptional activity of the *Hmgxb4* gene. We anticipate this *Hmgxb4* gene trap mouse line, as a reporter, will provide a valuable tool to recapitulate the expression level of endogenous *Hmgxb4* in physiological and pathological settings by measuring β‐galactosidase activity.

## 2 MATERIAL AND METHODS

### 2.1 Generation of the *Hmgxb4* gene trap mouse

The ES cell line RRO305 was obtained from BayGenomics (strain 129/Ola). The gene trap vector (pGT2Lxf) was inserted in the *Hmgxb4* gene between exons 5 and 6. The ES cells were injected into C57BL/6 blastocysts, which were then implanted into pseudo‐pregnant foster mothers. High‐contribution chimeras were obtained. After obtaining germ line transmitted mice, the heterozygous mice were backcrossed to C57BL/6J mice for at least eight generations and kept in C57BL/6J background. The use of experimental animals is approved by the Institutional Animal Care and Use Committee at Augusta University. The *Hmgxb4* gene trap mice described in this study will be available to the research community upon request. Embryonic day (E) 0.5 was defined as noon of the day when the vaginal plug was detected.

### 2.2 DNA extraction, genotyping by PCR (polymerase chain reaction)

DNA was extracted from tail biopsy essentially following the manufacturer's protocol (Viagen Biotech, Cat. No. 101‐T). Genotyping by PCR (ThermoFisher Scientific, Cat. No. K1082) were performed using the primers listed below as we previously described (Wang et al., 2014). PCR products were then run onto the agarose gel and visualized under UV after staining with ethidium bromide.

### 2.3 Protein extraction and western blot

Brain or aortic tissues were harvested from 4‐month‐old WT and their littermate *Hmgxb4* homozygous mice as we previously described (He et al., 2021; Wen et al., 2017, 2019). Tissues were ground with a glass homogenizer in 100 μL RIPA buffer (ThermoFisher Scientific, Cat. No. 89900) with 1% proteinase (ThermoFisher Scientific, Cat. No. A32963) and phosphatase inhibitor cocktail (ThermoFisher Scientific, Cat. No. A32957). After sonication and centrifugation of the lysate, proteins were quantified by BCA assay (ThermoFisher Scientific, Cat. No. 23225) and then loaded in a 9% SDS‐PAGE gel at 10–20 μg per lane. Antibodies used in this study are: HMGXB4 (Sigma, HPA000725, rabbit, 1:1000), GAPDH (Santa Cruz, V‐18, sc‐20357, goat, 1:2000), MYH11 (Sigma, M7786, mouse, 1:2000), MYLK (abcam, ab76092, rabbit, 1:2000), TGFB1I1 (Hic5, BD Transduction Laboratories, 61164, mouse, 1:3000), CNN1 (abcam, ab46794, rabbit, 1:5000), TAGLN (abcam, ab10135, goat, 1:3000), VCL (Sigma, V4505, mouse,1:5000). All HRP‐conjugated secondary antibodies were purchased from Abcam (Goat anti‐mouse IgG, Cat. No. ab205719; Goat anti‐rabbit IgG, Cat. No. ab205718; Donkey anti‐goat IgG, Cat. No. ab205723). Western blot images were acquired by ImageQuant LAS 8000 Imaging Station (GE).

### 2.4 Whole mount X‐gal staining

Whole mount X‐gal staining for mouse embryos or tissues were carried out as we described previously (Wen et al., 2017). Briefly, E10.5 embryos were dissected out from uterus or tissues were isolated from 4‐month‐old adult mice followed by fixing with 4% paraformaldehyde in PBS (phosphate buffered saline) for 30 min on ice. After rinsing the fixed embryos or tissues for three times with PBS for 15 min at room temperature, embryos or tissues were then incubated with 1 mg/mL X‐gal staining buffer (MilliporeSigma, Cat. No. B9146‐10MG) over‐night at 37°C in the dark. Subsequently embryos or tissues were rinsed with PBS, postfixed with 4% paraformaldehyde and taken photos under a dissecting microscope.

### 2.5 Mouse carotid artery ligation injury and β‐galactosidase activity measurement

Mouse carotid artery ligation was performed as previously described (Dong et al., 2021; Kumar & Lindner, 1997). Briefly, 10‐week‐old WT or *Hmgxb4* gene trap mice were anesthetized under Isoflurane. The left carotid artery (LCA) was dissected and completely ligated just proximal to the carotid bifurcation. The right carotid artery (RCA) served as an uninjured contralateral control. Following overdosed isoflurane mediated euthanasia and PBS perfusion from the left ventricle, the LCA and RCA were harvested 7 days after injury for western blotting or for assessing β‐gal activity using the Galacto‐Light Plus β‐Galactosidase Reporter Gene Assay kit (Invitrogen, Cat. No. T1007), essentially following the manufacturer's protocol. Twenty‐one days after ligation, the LCA and RCA were harvested for immunohistochemistry.

### 2.6 Immunohistochemistry

Immunohistochemistry assay was performed as described in our previous report (Wang et al., 2011). To detect HMGXB4 expression, LCA and RCA were harvested from WT mice post‐surgery 21 days and fixed in 4% paraformaldehyde, then embedded in paraffin. Paraffin embedded tissues were sectioned at a thickness of 8 μm and then stained with HMGXB4 (Sigma, HPA000725, 1:200) or ACTA2 (SM α‐actin; Sigma, A2547, 1:000) antibody. For detection of primary antibodies, we used avidin‐biotin method with diaminobenzidine substrate as chromogen (brown; Vector Laboratories; Cat. No. SK‐4100). Sections were lightly counterstained using hematoxylin to visualize cell nuclei (blue). Control sections were processed identically as experimental samples except no primary antibody was applied.

### 2.7 Preparation of mouse vascular smooth muscle cells (VSMCs) and adenoviral infection

VSMCs were prepared from 3‐ to 4‐week‐old WT or gene trap mice as we previously described (Ahmed et al., 2018; Dong et al., 2021). At the fifth passage, VSMCs were split for infection with adenovirus expressing *lacZ* for 48 h as described previously (Wen et al., 2019). X‐gal staining was performed as described as above.

### 2.8 Total RNA isolation and quantitative reverse transcription PCR (qRT‐PCR)

Total RNA from mouse tissues was extracted by TRIzol reagent (Invitrogen, Cat. No. 15596026) as we previously described (He et al., 2021; Wen et al., 2017). RNA (0.5–1 μg) was utilized as a template for RT with random hexamer primers using the High‐Capacity RNA‐to‐cDNA Kit (Applied Biosystems, Cat. No. 4387406). qRT‐PCR was performed with primers as described below. All samples were amplified in duplicate. In some cases, RT‐PCR product band after separated in agarose gel was purified by an agarose gel extraction kit (ThermoFisher Scientific, Cat. No. K210012) for Sanger sequencing. All sequencing was performed by Genewiz.

### 2.9 Oligonucleotides for RT‐PCR or qRT‐PCR

P1 (forward): 5′‐GAGAAGCGGCACTCCCGGACCAAG‐3′

P2 (reverse): 5′‐GACAGTATCGGCCTCAGGAAGATCG‐3′

P5 (forward): 5′‐CACAAGAAGAAGAGGAAGCACTCCC‐3′

P6 (reverse): 5′‐GGAGGTGATAGCTTTCAACAGGTCC‐3′

P7 (reverse): 5′‐CGGTACTCCTTACAGAATACCTGGTAG‐3′

P8 (reverse): 5′‐CAGGTAGTTGTGTGGTCAGGCATG‐3′

### 2.10 Oligonucleotides for genotyping PCR

P3 (forward): 5′‐TTATCGATGAGCGTGGTGGTTATGC‐3′

P4 (reverse): 5′‐GCGCGTACATCGGGCAAATAATATC‐3′

DNA control (forward): 5′‐CTAGGCCACAGAATTGAAAGATCT−3′

DNA control (reverse): 5′‐GTAGGTGGAAATTCTAGCATCATCC‐3′

### 2.11 Transthoracic echocardiography

Transthoracic echocardiography was performed with a linear 30 MHz transducer on 4‐month‐old mice using a VEVO 2100 echocardiography system (VisualSonics) as we previously described (Liu et al., 2021). Left ventricular (LV) morphometric and functional parameters were analyzed off‐line using VEVO 2100 software. M‐mode tracings were used to measure LV posterior wall thicknesses at end diastole (LVPW;d) and at end systole (LVPW;s). LV internal diameter (LVID) was measured as the largest anteroposterior diameter in either diastole (LVID;d) or systole (LVID;s). The LV ejection fraction (EF) was calculated with the modified Teicholz formula. LV fractional shortening (FS) was calculated according to the following formula: FS (%) = [(LVID;d − LVID;s)/LVID;d] × 100. The data were analyzed by a researcher blinded to the mouse genotype.

### 2.12 Tissue embedding, sectioning, and Masson's trichrome staining

For Masson's trichrome staining, hearts from 4‐month‐old adult mice were fixed with 4% paraformaldehyde over‐night at 4°C, and then embedded in paraffin. Sections were cut at 8‐μm thickness. Heart morphology and fibrosis were determined by Masson's trichrome staining (American MasterTech Scientific, Cat. No. KTMTRPT) following the manufacturer's instructions as we recently described (Liu et al., 2021). Images were acquired under 10× or 20× lens of an Olympus BX41 upright microscope. All images were composed in Adobe Illustrator software.

### 2.13 Blood pressure measurement by radio telemetry

The experiments were conducted in 4‐month‐old female and male mice as we previously described (Manhiani et al., 2015). Biotelemetry devices (PA‐C10, Data Sciences International) were implanted in the LCA in WT and *Hmgxb4* homozygous gene trap mice under isoflurane anesthesia. After recovery, mice were housed individually in a light‐ and temperature‐controlled room in the animal facility. Mice were given at least 3 days to recover from surgery before blood pressure measurements began. After 6 days of baseline measurement, an 11‐day Angiotensin II (Sigma, A9525) infusion was begun at 200 ng/kg/min using Alzet 1002 osmotic minipumps (DURECT, Cupertino, CA). Analog signals from the transmitters were sampled for 5 s every 2 min at 500 Hz, and the average of those measurements was recorded as the daily mean arterial blood pressure for each animal.

### 2.14 Statistical analysis

Data are expressed as means ± SD, and statistical analysis with Prism software (GraphPad). Differences with *p* < 0.05 were considered significant.

## 3 RESULTS AND DISCUSSION

### 3.1 Generation and genotyping of *Hmgxb4* gene trap mice

**FIGURE 1:**
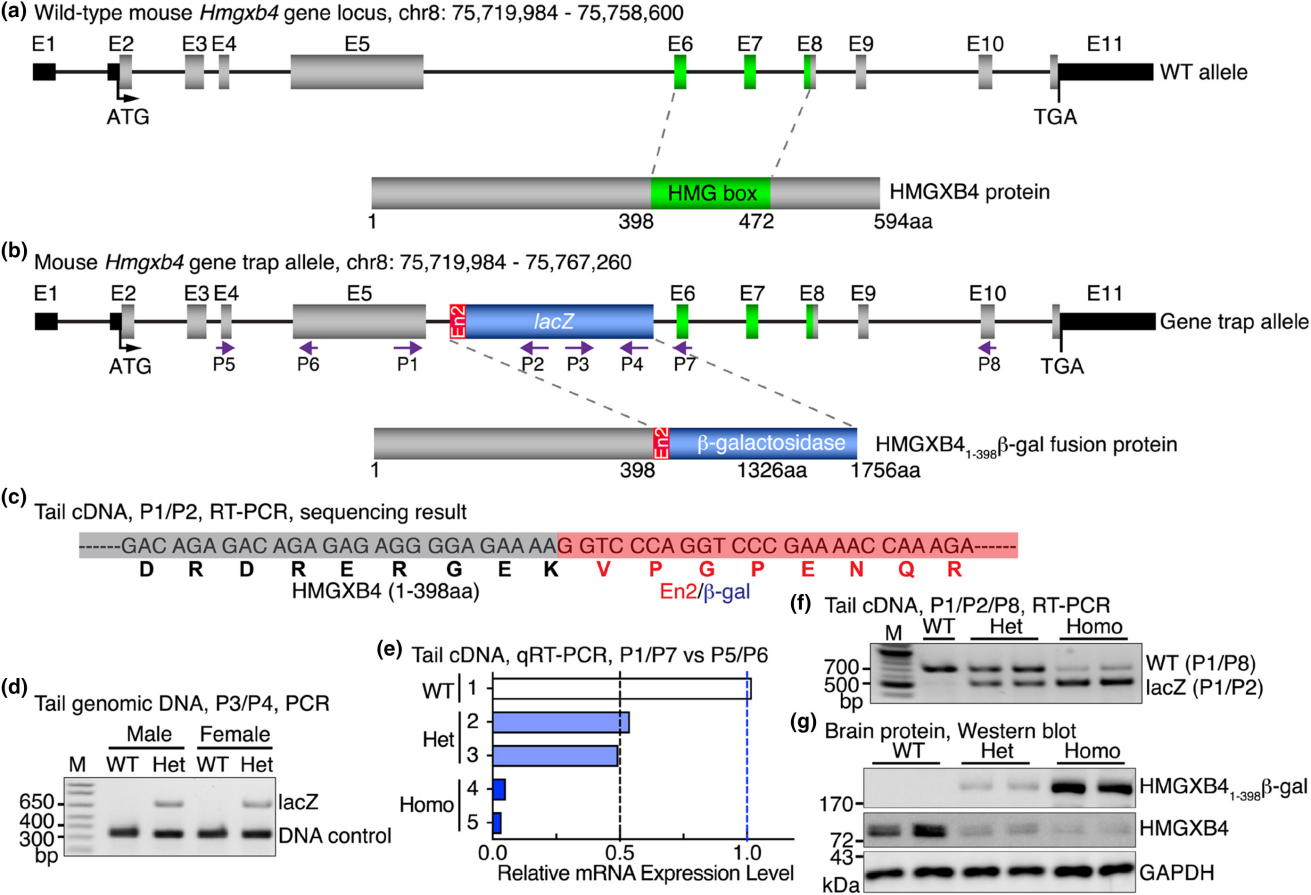
Generation and validation of *Hmgxb4* gene trap mouse. (a) Scheme depicting the gene structure of mouse wild‐type (WT) *Hmgxb4* gene which produces a 594 amino acid protein. The mouse *Hmgxb4* gene consists of 11 exons (E). The high mobility group (HMG) box domain (green) is encoded by the E6, 7, and partial E8. (b) Schematic diagram showing the gene trap vector pGT2Lxf insertion in *Hmgxb4* allele. The gene trap vector containing *lacZ* gene (encoding β‐galactosidase) is inserted into the intron between E5 and E6 of the *Hmgxb4* gene. Due to the presence of En2 (Engrailed, red) splice acceptor at the 5′ of gene trap vector, the insertion results in a premature termination of full‐length *Hmgxb4* expression following E5, thereby producing a fusion protein containing the N‐terminal 398aa residues of HMGXB4 and β‐galactosidase (HMGXB4_1‐398_β‐gal). Arrows (in purple) are the primers (P) designed for characterization of the trap allele. (c) DNA sequence of the linking region between *Hmgxb4* E5 and En2/β‐gal by sequencing cDNA from adult heterozygous mouse tail with primers located in E5 and *lacZ* (P1 and P2) as described in “b”. (d) Genomic DNA was extracted from adult mouse tail biopsies of offspring of WT crossed with heterozygous (Het) *Hmgxb4* gene trap mice and then PCR was performed with a pair of primers (P3/P4) that reside within *lacZ* gene trap cassette (680 bp). PCR for detecting a single copy gene *Il2* was served for DNA input control (324 bp). M: DNA marker. (e) Total RNA was extracted from tails of five adult offspring mice of intercrossed heterozygous (Het) *Hmgxb4* gene trap mice and reverse‐transcribed to cDNA. qRT‐PCR was then performed to quantify the expression of *Hmgxb4* mRNA by the primer sets (P1/P7 and P5/P6) that depicted in “b”. The relative level of expression of *Hmgxb4* by each of primer set was then plotted as 2^−(P1/P7 CT−P5/6 CT)^. (f) cDNA was prepared from adult WT, heterozygous (Het) and homozygous (Homo) mouse tail and then PCR was performed with the primers P1/P2/P8 as indicated. (g) Protein was extracted from brain tissues of adult WT, heterozygous (Het) and homozygous (Homo) *Hmgxb4* gene trap mice and Western blotting was then performed to examine HMGXB4 protein expression as indicated. GAPDH served as the loading control.

As the first step toward determining the expression of *Hmgxb4* in mice, we generated a novel *Hmgxb4* gene trap mouse model by using the *Hmgxb4* gene trap embryonic stem (ES) cell line RRO305 (BayGenomics) (Stryke et al., 2003) which contains a pGT2Lxf gene trap vector that integrates into the *Hmgxb4* intron (~20 kb) between the exon (E) 5 and exon 6 (Figure 1a,b). Due to the En2/splicing acceptor in the 5′ of *lacZ* cassette, the insertion disrupts full‐length *Hmgxb4* gene expression by splicing E5 of *Hmgxb4* gene to the *lacZ* cassette, resulting in a fusion transcript containing the E1–5 of *Hmgxb4* and En2/*lacZ* (Figure 1b). The original *Hmgxb4* gene trap ES cells were from mouse strain 129 and were used to generate chimeras by injection into C57BL/6 blastocysts. The resulted germline transmission mice were then backcrossed eight generations with C57BL/6J mice to obtain *Hmgxb4* heterozygous gene trap mice in C57BL/6 background. As predicted, sequencing of cDNA that from the heterozygous *Hmgxb4* gene trap mouse tail revealed that the *Hmgxb4* E5 (in gray) and En2/β‐gal (in red) are in the same reading frame, producing a fusion protein containing N‐terminus of HMGXB4 (1‐398aa) and En2/β‐galactosidase encoded by the *lacZ* (Figure 1c). Although the site of integrated gene trap vector was not identified due to the length and complexity of genomic DNA, we can readily detect gene trap allele in the heterozygous mice to distinguish from wildtype (WT) mice by directly PCR *lacZ* cassette (primers P3/P4) (Figure 1d). Furthermore, as *Hmgxb4* is widely expressed in mouse tissues including tail, we successfully developed a genotyping method by using qRT‐PCR to assess *Hmgxb4* mRNA expression in mouse tail. We designed the primers P1/P7 or P5/P6 to detect *Hmgxb4* E5–6 or E4–5 transcripts, respectively, to distinguish prematurely terminated mutant transcripts from WT transcripts (Figure 1b). These primers were then utilized to assess the *Hmgxb4* mRNA expression in mouse tails from offspring of intercrossed heterozygous *Hmgxb4* gene trap mice by qRT‐PCR. Data from this experiment revealed that heterozygous *Hmgxb4* gene trap mouse tail expresses approximate 50% while homozygotes (Homo) express about 3% or undetectable *Hmgxb4* mRNA relative to WT (Figure 1e). Intercrossing of heterozygous *Hmgxb4* gene trap mice produced WT, *Hmgxb4* heterozygous and homozygous mice with expected Mendelian distribution. All *Hmgxb4* Homo gene trap mice are viable and fertile, and appear normal without any gross physical or behavioral abnormalities.

To simplify the genotyping protocol, we re‐designed the primers (P1/P2/P8) with which can readily distinguish WT from gene trap transcripts by regular RT‐PCR. Using the cDNA isolated from adult mouse tail biopsy as the template, the agarose gel picture showed a clear separation of WT and E5/*lacZ* hybrid transcripts and a quantitative difference between heterozygous (Het) and homozygous (Homo) gene trap mice (Figure 1f). Western blotting with adult mouse brain tissues demonstrated that expression of endogenous HMGXB4 protein in Homo gene trap mouse was mostly disrupted, leading to a fusion protein of truncated N‐terminus HMGXB4 (1–398 aa) with β‐galactosidase (β‐gal). The heterozygous mice co‐express both HMGXB4_1‐398_β‐gal fusion protein and WT HMGXB4 protein simultaneously, suggesting biallelic expression of *Hmgxb4* gene (Figure 1g). The incomplete deletion of *Hmgxb4* in homozygous gene trap mouse tissues suggested that the gene trap cassette did not completely abolish *Hmgxb4* transcription, as such a minor fraction of WT transcripts can be produced by splicing around the inserted gene trap cassette. Taken together, these data demonstrate that the gene trap inserted into the mouse *Hmgxb4* gene locus leads to produce a fusion protein of N‐terminus HMGXB4 (1‐398aa) with β‐gal and the homozygous gene trap mice are viable.

### 3.2 X‐gal staining with tissues of *Hmgxb4* gene trap mice revealed HMGXB4 is widely expressed and is a nuclear protein

**FIGURE 2:**
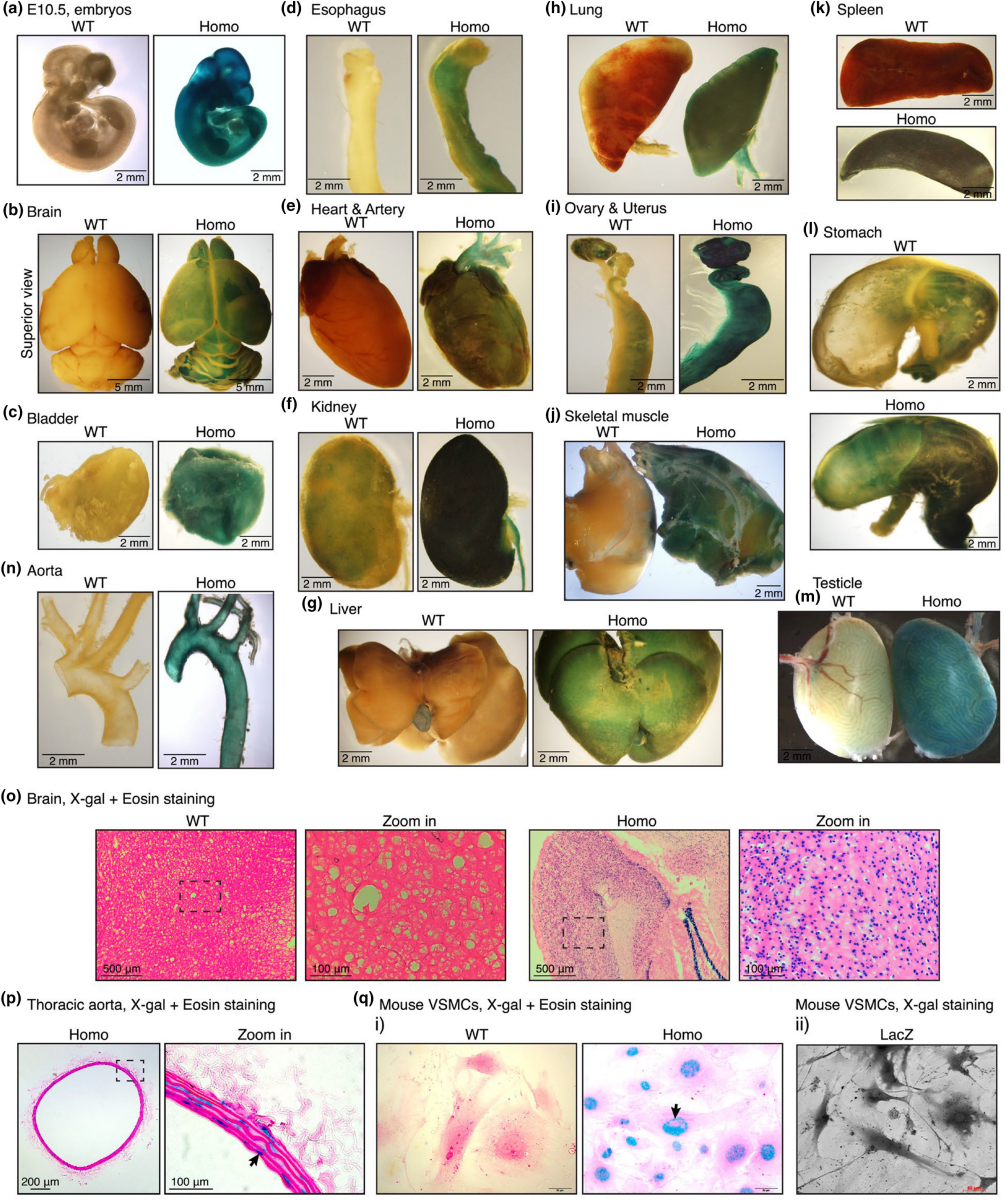
X‐gal staining of *Hmgxb4* gene trap mouse tissues. (a) E10.5 embryos from *Hmgxb4* heterozygous gene trap mouse intercrossing were collected at E10.5 for X‐gal staining. (b–n) X‐gal staining was performed with tissues harvested from adult WT or homozygous (Homo) *Hmgxb4* gene trap mice as indicated. (o, p) Sections from WT and homozygous (Homo) mouse brain (o) or dorsal aorta (p) was stained with X‐gal (blue) and counterstained with eosin (red). The boxed area is magnified to its right. Arrow indicates a representative X‐gal positive SMC in the media layer (p). (q) Aortic SMCs were prepared from WT or homozygous (Homo) *Hmgxb4* gene trap mice and stained with X‐gal (blue, arrow) and eosin (red) (i). X‐gal staining of mouse VSMCs infected with *lacZ* virus served as a control to show the localization of β‐gal expression in VSMCs (ii).

As the expression of HMGXB4_1‐398_β‐gal fusion protein is under control of the endogenous *Hmgxb4* gene promoter, X‐gal staining for β‐galactosidase activity can be used to examine the endogenous HMGXB4 expression. By using X‐gal staining in homozygous mouse embryos or adult mouse tissues, we found HMGXB4 expression is widely distributed throughout embryonic development and highly expressed in nearly all tissues examined (Figure 2a–n). X‐gal staining with sections of brain from homozygous mice confirmed that HMGXB4 expression is expressed in nuclei and widely expressed in almost every cell, especially in cells enriched in the hippocampus (Figure 2o). Consistently, X‐gal staining with sections of thoracic aorta further revealed that HMGXB4 expression is expressed in aortic SMCs (Figure 2p). X‐gal staining with cultured SMCs from WT or homozygous mice confirmed that HMGXB4 is indeed localized in SMC nuclei (Figure 2q‐i), as compared to the signal from *lacZ* virus infected SMCs (Figure 2q‐ii). Taken together, these data demonstrate that HMGXB4 is widely expressed in mouse tissues and is a nuclear protein.

### 3.3 *Hmgxb4* gene trap mice exhibit normal cardiovascular function

**FIGURE 3:**
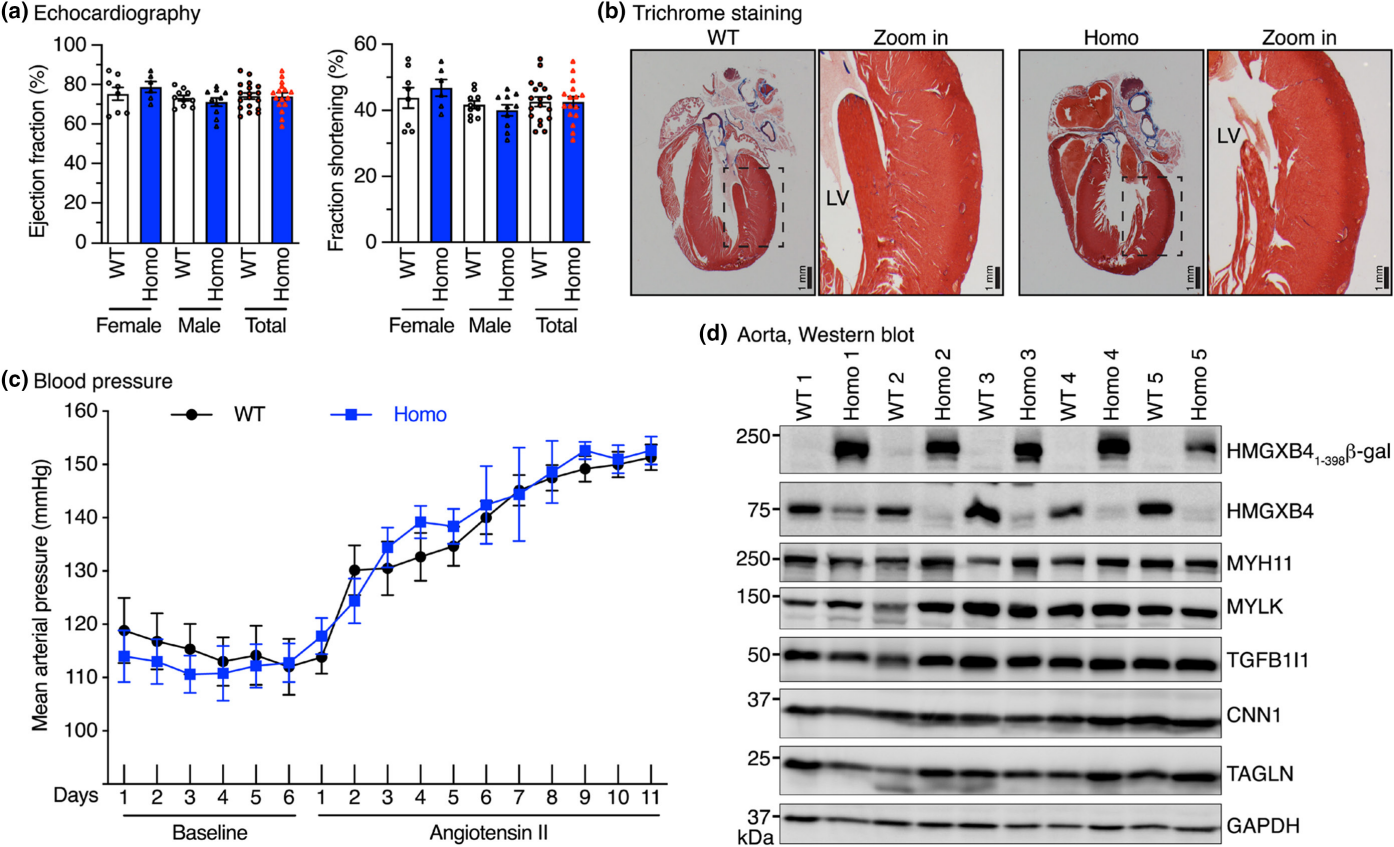
Characterization of the cardiovascular phenotype of *Hmgxb4* gene trap mice. (a) Echocardiographic quantification of cardiac function of adult WT (8 female, 10 male) and *Hmgxb4* homozygous (Homo) gene trap mice (6 female, 10 male) at 4 months of age. Shown are left ventricle ejection fraction (left panel) and fractional shortening (right panel). (b) Hearts from 4‐month‐old adult WT and *Hmgxb4* homozygous (Homo) gene trap mice were harvested for trichrome staining. The boxed area is magnified to the right. LV, left ventricle. (c) Mean arterial pressure of adult WT (*N* = 6, 3, males and 3 females) and *Hmgxb4* homozygous (Homo) gene trap mice (*N* = 5, 3 males, and 2 females) was measured 19 h/day by radio telemetry at baseline and during angiotensin II infusion. (d) A representative western blot indicates the comparable expression level of smooth muscle‐specific genes in aortic tissues harvested from WT (*N* = 5, 3 males, and 2 females) and *Hmgxb4* homozygous (Homo) gene trap mice (*N* = 5, 3 males, and 2 females).

Our previous study demonstrated that *Hmxb4* is a potent repressor that attenuates the key transcription co‐factor myocardin‐mediated smooth muscle myogenic function in vitro (Zhou et al., 2010). Since myocardin is specifically expressed in cardiomyocytes and smooth muscle cells (Wang et al., 2001), next we sought to characterize the potential cardiovascular phenotype of *Hmgxb4* gene trap mice. We first assessed the cardiac function of both male and female mice using echocardiography. Data from this measurement revealed that adult *Hmgxb4* homozygous gene trap mice had comparable EF and fraction shortening to that of WT control mice, regardless sex, indicating intact cardiac function (Figure 3a). Furthermore, trichrome staining showed normal heart structure and no signs of collagen deposition in the left ventricle wall in adult *Hmgxb4* homozygous gene trap mice (Figure 3b). Using a telemetry system to measure the blood pressure of mice in real time, we found no difference in the mean arterial pressure at baseline and after infusion with Angiotensin II between homozygous and WT control mice (Figure 3c). Moreover, there are no differences in the expression of smooth muscle‐specific proteins in thoracic aorta of homozygous and WT control mice (Figure 3d). Taken together, these data demonstrate that *Hmgxb4* gene trap mice exhibit normal cardiovascular function with normal contractile protein expression in their aortic tissues.

### 3.4 HMGXB4 expression is induced in response to vascular injury

**FIGURE 4:**
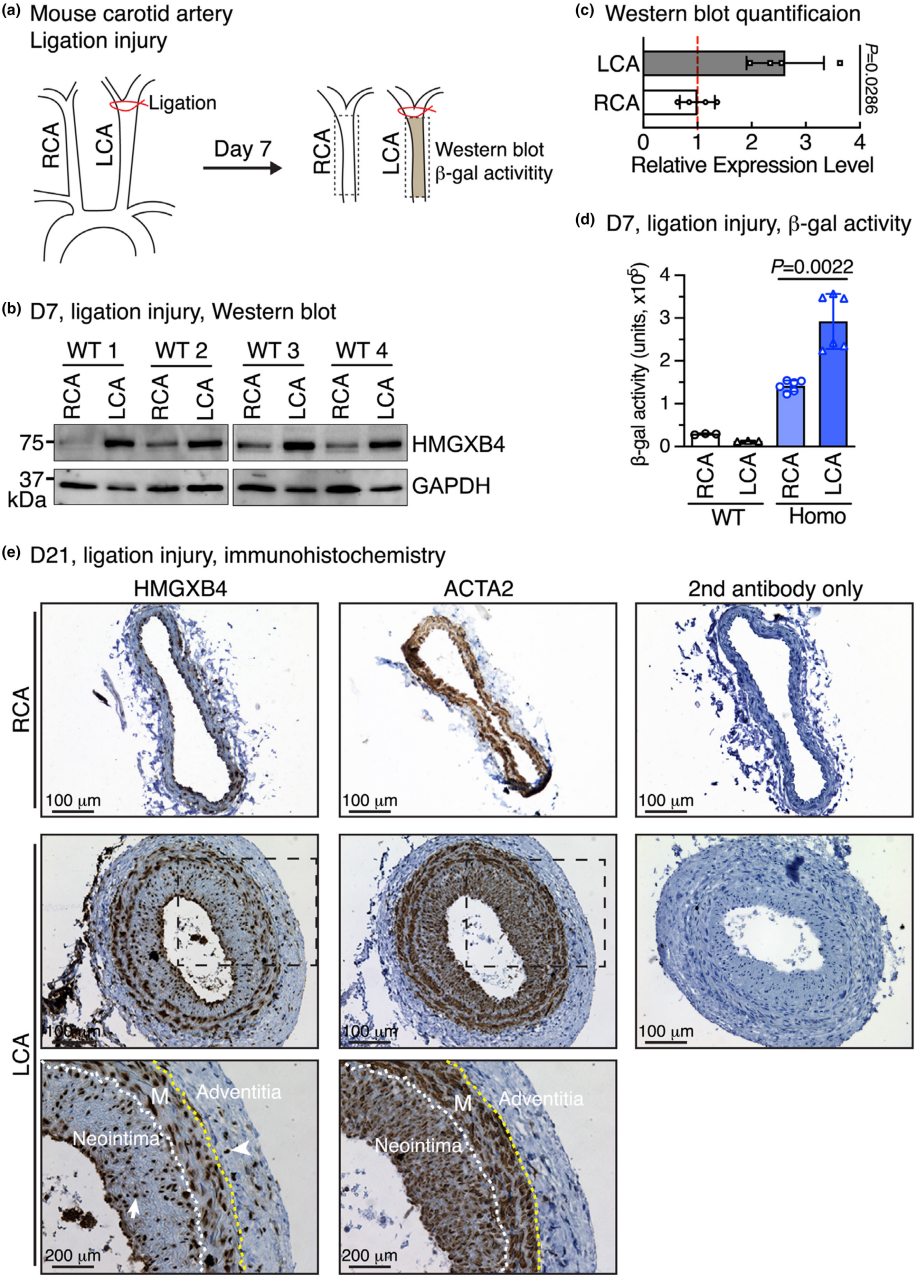
HMGXB4 expression is induced in response to vascular injury. (a) Schematic diagram showing the experimental design for the mouse carotid artery ligation injury. (b, c) At day 7 post ligation injury, the control right carotid artery (RCA) and the ligation‐injured left carotid artery (LCA) were harvested for western blotting to determine HMGXB4 expression. The band intensity of the blots shown in “b” was measured and the relative protein expression is plotted in “c”. *N* = 4. Unpaired Student *t*‐test. (d) Control RCA and the ligation‐injured LCA (Day 7) were harvested for β‐gal activity. *N* = 3–6. Unpaired Student *t*‐test. (e) Immunohistochemistry staining of HMGXB4 and the smooth muscle‐specific marker ACTA2 (brown) was performed on serial sections of 21‐day ligation injured left carotid arteries (LCA). Uninjured right carotid arteries (RCA) served as the contralateral control. Cell nuclei were stained by hematoxylin (blue). M: media smooth muscle layer. An arrow and arrowhead denote a representative neointimal and adventitial HMGXB4 positive cell, respectively.

Previous studies have shown that complete ligation of the vessel near the carotid bifurcation in mouse can induce rapid proliferation of medial smooth muscle cells, leading to extensive neointima formation (Kumar & Lindner, 1997). To test whether HMGXB4 expression is changed in response to vascular injury, first we examined HMGXB4 expression by western blot following mouse carotid artery ligation. We found in LCA on day 7 post arterial injury, HMGXB4 protein is induced, compared to the contralateral RCA (Figure 4a–c). As the expression of N‐terminal HMGXB4/β‐gal fusion protein is under control of the endogenous *Hmgxb4* gene promoter, measurement β‐galactosidase activity can be used as a reporter for quantitatively assessing the endogenous *Hmgxb4* gene expression. Therefore we next measured the β‐galactosidase activity following the carotid artery injury for 7 days in WT and *Hmgxb4* gene trap homozygous mice. Consistent with the western blot data, the β‐gal activity in the injured LCA is significantly elevated, compared to the uninjured RCA (Figure 4d). To determine which cell types contribute to the induced expression of HMGXB4 after injury, immunostaining of the smooth muscle‐specific marker ACTA2 and HMGXB4 was performed on serial sections of 21‐day ligated injured arteries. Data from this assay revealed that HMGXB4 is expressed in both neointimal and medial layer smooth muscle cells, in addition to some sporadic expression in adventitial cells in injured carotid arteries in mice (Figure 4e). This data suggests that the induced expression of HMGXB4 after injury mainly originates from smooth muscle cells. Taken together, this study suggest that the *Hmgxb4* gene trap mouse can be utilized to monitor *Hmgxb4* gene expression in pathological conditions in a quantitative manner.

In summary, the *Hmgxb4* gene trap allele described here results in a significant reduction of WT HMGXB4 protein expression and is a valuable genetic tool as a reporter to examine HMGXB4 expression in variety of mouse tissues under physiological and pathological settings. It should be pointed out this *Hmgxb4* gene trap allele may not be functionally null in vivo due to the remaining expression of large portion of N‐terminal HMGXB4 although we previously demonstrated that the NT HMGXB4 has no effects on myocardin‐induced transactivation on SM‐specific gene promoters (Zhou et al., 2010). The important role of *Hmgxb4* in cardiovascular system remains to be determined by using the conditional *Hmgxb4* mouse model we recently generated (He et al., 2021).

## FUNDING INFORMATION

The project was supported in part by a start‐up fund from Augusta University. Dr Zhou is a recipient of an Established Investigator Award (17EIA33460468) and Transformational Project Award (19TPA34910181) from the American Heart Association. Drs. Xiangqin He and Kunzhe Dong are supported by a postdoctoral fellowship (836341) and a career development award (938570), respectively, from the American Heart Association.

## CONFLICT OF INTEREST STATEMENT

None.

## ETHICS STATEMENT

The use of experimental animals and viral work is approved by the Institutional Animal Care and Use Committee and Biosafety Committee at Augusta University (protocol number: 2012‐0502) in accordance with the guidelines of the National Institutes of Health.

## Data Availability

The *Hmgxb4* gene trap reporter mouse line and all source data will be made available from the corresponding authors upon request.
